# A narrative review on problems in product quality, regulatory system constraints, and the concept of quality by design as a solution for quality assurance of African medicines

**DOI:** 10.3389/fmed.2024.1472495

**Published:** 2024-10-03

**Authors:** Hassen Kebede Hassen, Yesuneh Tefera Mekasha, Addisu Afrassa Tegegne, Yildiz Ozalp

**Affiliations:** ^1^Veterinary Drug and Feed Control Administration and Control Authority, Addis Ababa, Ethiopia; ^2^Pharmaceutical Sciences, Pharmaceutical Quality Assurance, and Regulatory Affairs, University of Gondar, Gondar, Ethiopia; ^3^Department of Pharmaceutical Technology, Faculty of Pharmacy, Near East University, Nicosia, Cyprus

**Keywords:** narrative review, poor quality medicine, challenges, quality by design, regulatory system, Africa

## Abstract

**Background:**

The provision of medicines with confirmed quality and efficacy is critical for maintaining the public health and building confidence in the healthcare systems. However, the presence of poor-quality medicines still presents a significant challenge in the pharmaceutical landscape across the African regions. This is further exacerbated by the lack of consistency or discrepancy in the current regulatory framework. As a consequence, given the current constraints, a robust regulatory structure that can guarantee the supply chains attainment of the intended medicinal product requirements are required.

**Objective:**

The review aimed to provide a detailed analysis of the quality issues in the pharmaceutical supply in Africa, highlighting the challenges and proposing potential solutions for its mitigation.

**Methods:**

The review was conducted from May 2023 to April 2024. This narrative review examined poor-quality medicines, regulatory challenges, and mitigation strategies in the African pharmaceutical industry. The review utilized databases such as Google Scholar, PubMed, and Web of Science. The search strategy was customized to include open-access articles published in peer-reviewed scientific journals in English and focused exclusively on studies conducted in African countries.

**Results:**

The review portrays the prevalence of poor-quality medicinal products in various regions of Africa. Among various categories of findings, 42% of the reports on poor-quality medicinal products come from the African region, as per the WHO report. Furthermore, separate findings on substandard medicinal products from many African countries were encountered. The presence of problems in the regulatory system, such as the absence of any pharmacopeia belonging to any African country and variation/inconsistency in each country’s regulatory set-up, was indicated. Other factors for the inability to enforce regulatory law, such as insufficient skilled and committed human resources, the presence of corruption, as well as financial resource scarcity, were revealed in the review. From the situational analysis, the possibility of building a robust quality assurance system in the near future through a quality by design approach under existing resource limitations was discussed.

**Conclusion:**

The pharmaceutical sector in Africa faces significant challenges, including the prevalence of poor-quality medicines and weak regulatory enforcement. Tackling these challenges are vital for enhancing health outcomes throughout the continent through the provision of high-quality medicines. Trending toward quality by design in the quality assurance system under prevailing financial scarcity can be very beneficial.

## 1 Introduction

Medicines require special attention, without which, many people around the world are denied proper healthcare (1). Unlike typical consumer products, even small deviations from the recommended dosage or formulation of medicines can have serious health impacts (2). This is because any excess or deficiency can lead to adverse effects or ineffective treatment (3). On the contrary, deviation from the theoretical recommendations of drug monograph can also bring either a lack of desired clinical outcome or the worst scenario of development of drug resistance in the case of antimicrobials (4). Guaranteeing fair access to safe and affordable medications is essential for attaining the highest possible standard of health, which aligns with one of the Sustainable Development Goals. To enhance the utilization of medicines and their beneficial effects on public health, it is imperative to develop a strong regulatory framework for pharmaceuticals (5). In Africa, the National Medicines Regulatory Authorities encounter multiple challenges. These include prolonged product registration timelines, underdeveloped regulatory structures, redundancy in regulatory procedures, shortages in organizational capacity, and inefficiencies in certain cases (6).

The need for special precautions in regulating the quality of medicinal products has been recognized for centuries. This is exemplified by the establishment of various institutions and regulations dedicated to ensuring the quality of medicine. One of the earliest examples is the creation of the United States Pharmacopeia by a group of volunteers in 1820 (7). The establishment aimed to compile a comprehensive collection of standardized recipes for drug preparation, ensuring consistency and dependability throughout the United States. Furthermore, the United States Drug Importation Act of 1848 formally acknowledged the United States Pharmacopeia as a credible authority on drug quality standards and inspection services. This legislation required the inspection of imported pharmaceuticals at customs to verify compliance with the defined quality criteria. It represented the inaugural law in the United States focused on regulating drug quality. By emphasizing the need to determine medications that meet requirements beyond pharmaceutical or biological quality, as well as demonstrating preclinical, and clinical safety as well as efficacy in medicinal products, this act laid the groundwork for modern drug regulation (8).

Despite the historical events with the establishment of medicines quality affiliated centers as early as the beginning of the 19th century, few documented evidences from Africa indicated the emergence of medicines quality regulation-related guidelines even until the end of the first half of the 20th century documented in Ethiopia (6). Unfortunately, none of the African countries, except Egypt, currently have their national pharmacopeia (9, 10). Though there is institutional awareness of worldwide regulatory frameworks, quality control has been based on stringent sampling and laboratory testing procedures, which are not financially or temporally practical. The modern regulatory landscape has moved beyond just “quality by testing” or “quality by chance” methodologies and has instead placed a singular emphasis on the principles of quality by design (11, 12). It was first coined in the USA since in 1992 and becomes an institutionalized regulatory concept in FDA at year 2004 GC (13, 14). Nowadays quality by design paradigm is advocated for its efficiency in terms of time and cost.

The study revealed that, due to poorly implemented regulatory frameworks for medicines in Africa, there has been evidence of the influx of substandard and falsified medicinal products into the continents (15). For instance, the study conducted in 2024 indicated that 22.6% of poor-quality drugs were found in Africa (16). Additionally, about 34.6% medicine found in the market were unregistered (17). This report emphasizes how critical it is to solve the problems caused by counterfeit and substandard medications in Africa. To ensure the availability of safe, effective, and high-quality medications, strengthening regulatory mechanisms, building capacity, encouraging collaboration, and raising public knowledge are essential initiatives that will preserve public health and rebuild public confidence in healthcare systems (18).

The prevalence of substandard medicines in Africa can be attributed to the inadequate and flexible regulatory frameworks governing the pharmaceutical industry. To mitigate this issue, it is essential to establish stringent quality standards and design principles. Addressing the historical oversight of quality in African nations is vital, necessitating continuous efforts to enhance regulatory measures. Consequently, the pharmaceutical sector in Africa should focus on integrating quality into the product development process from the beginning, rather than relying exclusively on end-stage testing (19). Without adherence to these principles, it becomes challenging to address problems effectively and implement corrective and preventive actions. Accordingly, developing the concept of quality by design (11) in a pharmaceutical environment is critical as a solution of quality assurance of pharmaceutical products. This will enable more flexible regulatory relief, while still guaranteeing product quality and patient safety.

According to a report by the World Health Organization, substandard and falsified medicinal products represent a significant global issue that endangers public health and patient safety, while also contributing to a concerning rise in antimicrobial resistance. This issue is particularly widespread in low- and middle-income countries, where it is estimated that one in ten medical products may be substandard or falsified (20). Evidence suggests that the presence of substandard antimicrobials in Africa was unavoidable; nevertheless, there is often a failure to recognize the fundamental reasons for quality deficiencies in the manufacturing area (16, 21). The principle of quality by design is not yet widely implemented in contemporary pharmaceutical manufacturing facilities in Africa. In the current scenario, the target product quality profile is one of the critical elements of quality by design in regulatory environments. The TPP will help identify critical quality attributes such as potency, purity, bioavailability or pharmacokinetic profile, shelf-life, and sensory properties (22).

This review has been undertaken to identify current problems in medicinal product regulation in Africa and forward solutions for efficiency improvement options through a comparative quality by design approach. The review focuses on documenting defective products within the categories of agricultural pesticides, veterinary drugs, and human medicinal products. Moreover, it aimed to identify factors contributing to the presence of defective products in the market and propose QBD-based alternatives as remedies for existing problems.

## 2 Materials and methods

### 2.1 Search strategy

The narrative review, conducted from May 2023 to April 2024, focused on examining the issue of substandard pharmaceuticals, challenges, and potential mitigation strategies within Africa’s pharmaceutical environment. The review utilized databases such as Google Scholar, PubMed, and the Web of Sciences. Key steps and methodologies involved in the review were language and time frame, which were restricted to English-language publications, drug advocacy websites, and data collected from African published literature that specifically addressed issues related to poor-quality medications, existing problems with regulatory standards, and potential solutions within the pharmaceutical industry on the continent.

### 2.2 Quality data evaluation method

The Medicine Quality Assessment Reporting Guidelines checklist was utilized to ensure the quality and rigor of the selected articles in the review. This checklist provides a structured framework for assessing the methodology of studies on medicine quality, encompassing 12 specific criteria (23) (Supplementary File 1). The included quality medicine articles had the following information: study objective, study design, sampling method, data collection, quality control tests, statistical analysis, ethical considerations, limitations of results reporting, interpretation of results, funding and conflicts of interest, and conclusion.

A comprehensive approach to revising the regulatory system information conducted by utilizing an online database and incorporating previously published findings ensures that the review was grounded in credible sources and up-to-date data (6, 24–27).

## 3 Literature search results

### 3.1 Historical evolution of medicines regulation

Since medicine has been a part of human history for centuries, methods for ensuring its quality have developed steadily throughout a time (28). Historically, the evolution of medicines regulation has been driven by the need to protect public health and ensure the safety and efficacy of pharmaceuticals (29). Unfortunate events, rather than the growth of medical knowledge, have been the main force behind the regulation of medicine. Risks are inherent in pharmaceutical a procedure, which emphasizes how important strong control is. Good regulation guarantees that pharmaceuticals, especially veterinary medications, fulfill quality, safety, and efficacy requirements (30). This is essential to preserving market integrity, safeguarding public health, and avoiding problems like inferior or fake goods from reaching consumers. Enforcing conformity with established recommendations and standards is a major responsibility of regulatory organizations. This entails carrying out routine inspections, keeping an eye on unfavorable incidents, and taking appropriate corrective action as needed. Regulators can contribute to the safe and efficient operation of the pharmaceutical business by upholding strict oversight and regularly updating standards in light of new information and developing dangers (31).

The evolution of medicine regulation has been complex, with significant milestones like the Apothecary Wares, Drugs, and Stuffs Act of 1540 and the Food and Drugs Act of 1875, and the National Medicines Regulatory Authority in the UK (32). Similarly, although different institutional naming, enactments for establishment NMRA’s in Turkey (33), Switzerland (1900), USA (1906), Norway (1928), and Sweden (1934) mainly for patent protection and trade promotion, though the laws in Norway and Sweden focused on product safety as well (34). Profession known to act starting from 1911 with further improvement to the Scientific Expert Committee of the German Medical Association (1958-61), and later on with official enactment passed in 1963 to establish the First German Medicines Act initiated in response to the thalidomide birth defect tragedy in 1961 (35).

In the USA, the official regulatory structure traces back since to the development of the pure foodPure Food and drugs act Drugs Act of 1904 by the US congress Congress. This is followed by the issuance of the Food, Drugs and cosmetic Cosmetic Act, of 1938 issued after the death of over 100 people in 1937 due to sulphanilamide elixir prompting assessment of safety before any product is marketed (36). In the early 1960s, the thousands of pregnancies were affected by thalidomide-induced phocomelia. It has also and other defects causing caused to transform and institutionalize institutionalized throughput drug safety and efficacy screening procedure establishments in NMRAs globally for investigational new drugs and monitoring of clinical trials has also received attention (32). The current European Medicines Agency was established in 1995 to ensure the safety and efficacy of medicine and medical devices within the modern-day 25-state member community (37). Pharmaceutical companies in today’s competitive environment employ diverse strategies to gain regulatory relief, whether through traditional methods like quality testing or systematic approaches. The traditional regulatory evaluation system assesses product quality and performance through constraints on manufacturing processes and final product testing. In contrast, modern regulations prioritize the incorporation of quality through design. Consequently, the present emphasis on quality implementation in pharmaceutical industries can be attributed to the principle of quality by design. The quality by design (11) concept concerning pharmaceutical quality assurance becomes an issue with its efficiency and effectiveness in terms of both time and money over routine quality assurance through rigorous sample analysis. In this regard quality by design is defined as building quality in design instead of testing from final product (38).

### 3.2 Current trends in medicinal product quality in African countries

Global sustainable development goals: The third priority goal of the global sustainable development program is to ensure healthy lives and promote well-being for all at all ages. This includes a focus on access to quality, safe, effective, and affordable essential medicines and vaccines (39). Among sub-targets considered, access “to quality, safe, effective and affordable essential medicines and vaccines for all is emphasized” (40).

**FIGURE 1:**
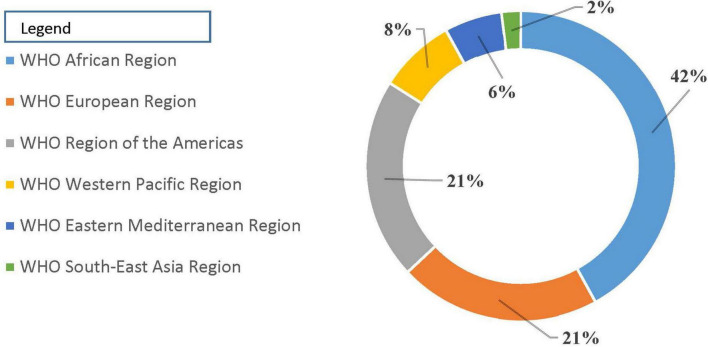
Reports of medicine counterfeited/substandard [Source: WHO (44)].

Challenges driving poor-quality medicines in Africa: Ensuring quality, safety, and effectiveness in the global medicine supply chain is fraught with challenges. In the African context, these challenges are compounded by limited financial resources, which impact the ability to access quality medicines. Apart from that, difficult and complex regulatory frameworks can impede the efficient distribution of quality medicines (41). Additionally, there is a higher prevalence of defective medicines due to gaps in regulatory implementation and poorly designed disincentives for non-compliance (42). This situation contributes to adverse health outcomes due to gaps in regulatory implementation as well as poorly designed disincentives for noncompliance, creating undesired health outcomes (43). A regional summary from the WHO revealed 42% of reports on defective quality medicines coming from the African continent (44) (Figure 1). Addressing the issue of falsified and substandard medicines in Africa also necessitates strengthening the local regulatory system for controlling pharmaceutical manufacturing practices. This local manufacturing can serve as a means to increase the availability and accessibility of quality essential medicines across the African continent.

#### 3.3 Consequences of defective medicinal product quality

Health impact: Unexpectedly High Active Pharmaceutical Levels: Medicines with unexpectedly high levels of the active pharmaceutical ingredient (45) can lead to toxic reactions, severe side effects, or even death (45, 46). Apart, contamination with harmful substances can cause serious health issues, including infections, organ damage, or cancer.

Long-term illness: Defects in medicinal product quality are attributed to life-threatening illnesses and other indirect socioeconomic outcomes. Either unexpectedly high levels of the expected API or else product contamination with other dangerous substances can result in death or long-term illness for individuals taking these defective quality medicines. Long-term illness may also be due to the continuation of the treatable disease that remained due to the absence or reduced level of API; perhaps a preventable disease can also rise in a community, especially in the case of defective vaccines. Defective-quality products with subtherapeutic API levels also pose a risk of antimicrobial resistance, resulting in the nullification of the role of antimicrobials for human survival (47, 48).

Indirect socioeconomic outcomes: The socioeconomic impact of defective product quality in the supply chain has been estimated. Globally Falsified medicinal products constitute a market share estimated to be US$ 200 billion making it the most profitable business among illegally copied items (49). In Africa, compared to other global perspectives, the rise of poor quality medicines was indicated attributable to the absence of strict supply chain regulation, track and trace technology as well as enforcement regimens that are in place in Europe and united states (40). Furthermore, the socioeconomic impact of defective quality medicines in Africa has also been estimated. Antimalarial drugs in sub-Saharan Africa resulting socioeconomic impact specifically deaths due to poor quality (47) of drugs estimated at 7,500–150,800 from malaria and pneumonia (50).

### 3.4 Prevalence of poor-quality medicine in African countries

The quality defects in the pharmaceutical market, particularly in Africa, are categorized into substandard, unregistered, falsified, and counterfeited products. Each category has distinct implications for regulatory management and public health (40). For reasons of avoiding disparity the World Health Organization has adopted a working definition of substandard medicinal products to refer to apparently authorized medicinal products that fail to meet quality standards for manufacturing and distribution, while unregistered or unlicensed products are products that have not passed through approval procedures by regulatory bodies before marketing. The last category, falsified or counterfeited products, is agreed to refer to products deliberately concealed or lied about in terms of product identity, composition, or source (51). The later classification was the most unreliable and unethical pharmaceutical trade, constituting a criminal act. Quality defects encountered in African pharmaceutical supply are thus discussed using the WHO working definition.

Under ideal circumstances, is it in Africa or elsewhere in other parts of the world that products in the pharmaceutical supply system should pass through national regulatory checkups for their quality, safety, and efficacy? However, the circulation of unregistered medicinal products has been reported in many African countries. Surveys highlighted the circulation of unregistered drugs in the supply chain in Ethiopia, mainly in border regions, due to weak border control and regulatory implementation (6). In Kenya, a survey on first-line antiretroviral drugs revealed that 27.47% of the products encountered were unregistered after being manufactured by known and licensed manufacturers. However, it was found with proper API content (52).

The common quality defect documented in African countries is the prevalence of substandard drugs failing to fulfill defined manufacturing standards such as assay, uniformity of packaging, labeling consistency, and product active matter release performance (23, 53–55). The most common defects in quality reported from the African drug supply chain consist of antimalarials, anti-infectives, and, to a lesser extent, antihelmentics (53, 56).

Estimating the proportion of counterfeit drugs in the pharmaceutical supply in African countries is challenging due to the limitations of convenience sampling, which often does not provide a comprehensive or representative picture. Despite these challenges, several studies have highlighted the prevalence of substandard and counterfeit drugs, particularly antimalarials, across various countries (57). In African countries, studies have been undertaken on the circulation of substandard and counterfeited drugs. In Ethiopia, quality analysis on ten veterinary product batches from six trademarks of Albendazole demonstrated two products failing to meet the minimum content as claimed (58). In Nigeria, studies on antibiotic drug products 48% (59); of samples of Amoxicillin, Amoxicillin-Cloxacillin combination-trimoxazole and Tetracycline and 36.5% (55) of samples of chloroquine and selected antibacterial drug were substandard compared to set pharmacopeias limits.

A counterfeit antimalarial drug product, Coartem^®^, was discovered in Cameroon in 2013 and distributed in West and Central Africa. The counterfeited product, known to contain little or no active pharmaceutical ingredient, was distributed in hospitals and street vendors, with the same logos as the Global Fund Affordable Medicines Facility—Malaria Programme in Cameroon and Nigeria (60). Recent reports on other counterfeited antimalarial drugs were also found documented from sub-Saharan African countries Uganda and Central African Republic (61), Cameroon, Chad, Democratic republic of Congo, Niger, Nigeria (62). Six batches of quinine sulfate 300 mg were found counterfeited from Chad (three), Cameroon (63), and Nigeria (63), with the manufacturer’s claim being Remedica Ltd., with no active ingredient detected in them.

The review highlighted illustrates significant challenges in the fight against counterfeit pharmaceuticals and diagnostic products:

Outdated WHO essential drugs program logo: The use of outdated or fraudulent logos on falsified products can mislead healthcare professionals and patients about the authenticity and quality of the medications. This underscores the need for vigilance and verification of the legitimacy of pharmaceutical products (64).

Falsified chloroquine phosphate tablets: The documentation by the World Health Organization (65) of falsified chloroquine phosphate tablets from Cameroon, the Democratic Republic of Congo, and Niger highlights the global nature of the counterfeit drug problem. Counterfeit chloroquine can undermine treatment efforts and pose serious health risks (66).

Counterfeit HIV diagnostic tests: The case of falsified Uni-Gold™ HIV tests from Kenya, with altered expiry dates, reveals the risks associated with counterfeit diagnostic tests. The discrepancy in expiry dates can lead to false results and inadequate treatment, further complicating the management of HIV (62). These instances emphasize the importance of robust regulatory frameworks, stringent quality control measures, and effective surveillance systems to combat counterfeit medicines and diagnostic products. Collaboration between regulatory authorities, manufacturers, and international organizations like WHO is crucial to address these challenges and protect public health (67).

A study from ten West African countries on cardiovascular drugs Amlodipine and Captopril from both licensed and illegal outlets has indicated a 50% prevalence of poor quality products among products from Asia in illegal outlets (68, 69). Drug quality differences for products with the same origin but different marketing region was also reported from product quality analysis in south Africa compared to other European country (70). The Substandard and Falsified products reported from African countries through Global Surveillance and Monitoring System were summarized in Table 1, and Substandard Medicinal Product Survey Undertaken and Published from Different African Countries were also summarized in Table 2.

**TABLE 1:** Substandard and falsified products reported from African countries through GSMS.

Drug entity	Country of product encounter	Physical form	Information labeled on the product			Quality analysis findings	Stated manufacturer	References
			Batch	Manuf. date	Expiry date			
Chloroquine phosphate	Cameroon	Tablets 100 mg	660	05/2017	05/2021	Falsified label and or contain below expected API	Jiangsu Pharm.	(62)
		Tablet 250 mg	660	09/2022	09/2022		Jiangsu Pharm.	
		Tablet 250 mg	660	05/2019	04/2023		Jiangsu Pharm.	
		Tablet 100 mg	660	08/2018	08/2022		Jiangsu Pharm.	
		Tablet 100 mg	EBT 2542	01/2019	10/2022		Astral pharm.	
Chloroquine phosphate	Democratic Republic of Congo	Tablet 250 g	N°:1605059	05/2019	04/2023		Dawa Limited Brown & Burk Pharm	
			N°: 065622	11/2018	11/2022			
Chloroquine phosphate	Niger	Tablet 100 mg	HV1116	06/2019	05/2023		None	
		Tablet 100 mg	NBJT02	11/2019	10/2022		None	
		Tablet 100 mg	NBJT01	11/2019	10/2022		None	
Uni-Gold HIV 1/2 rapid diagnostic kit	Kenya	KIT	HIV7120026		5/12/2020	Falsified labeling, delayed result	Trinity Biotech plc.	
			HIV6120030		29/07/20			
Quinine sulfate	Nigeria	Tablet 300 mg	44680	04/2017	04/2021	No quinine identified	REMEDICA LTD–Cyprus	
			44680	09/2017	10/2020			
Quinine sulfate	Chad	Tablet 300 mg	44680	10/2018	10/2023	No quinine identified; Traces of chloroquine	Remedica Ltd–Cyprus	
			44680	04/2017	04/2021			
			44680	03/2015	03/2018			
Quinine sulfate	Cameroon	Tablet 300 mg	44680	09/2017	10/2020	No quinine identified	Remedica Ltd–Cyprus	
Quinine sulfate	CAR	Tablet 300 mg	7711006	8/2018	7/2021	Falsified Label	Phamachim Bulgaria* and Enitop Pharmaceutical Nig. Ltd.# Phamachim, Bulgaria*	(61)
		Tablet 800 mg	00952005	06/2015	12/2020			
Quinine sulfate	Chad	Tablet 300 mg	7711004	5/2018	4/2021	Falsified Label	Phamachim Bulgaria and Enitop Pharmaceutical Nig. Ltd.	
Quinine bisulphate	Uganda	Tablet 300 mg	7422	03/2017	04/2021	Falsified label and no expected API	Laboratory & Allied Ltd.	
Augmentin (Amoxicillin trihydrate—Potassium clavulanate)	Uganda and Kenya	Tablet (500 mg/125 mg)	786627	Aug 2016	Aug 2019	Falsified Labeling and none of the API	SmithKline Beecham Limited	
Hydrochlorothiazide	Cameroon	Tablet 50 mg	16G04	06/2017	30/5/2021	Falsified labeling, No expected API@	Laboratoires Sterop	
Artemeter 20 mg + lumefantrine 120 mg combination	Cameroon	Tablet	NOF 2153	01.2013	11. 2015	Assay with very little or no API	100%	(60)
			F2929	01.2012	01.2016		100%	
			F1901	01.2012	01.2014		100%	
			F2261	01.2012	01.2014		100%	

@5gm glibenclamide trace obtained instead of expected hydrochlorothiazide, GSMS, Global Surveillance and Monitoring System. # Manufacturer name is indicated on the product label of declared counterfeited product. * Country of origin is indicated on the product label of declared counterfeited product (not necessarily).

**TABLE 2:** Substandard medicinal product survey undertaken and published from different African countries.

Drug entity	Place of encounter	Dosage form	Information Labeled on the product			Quality analysis findings	Proportion of poor quality sample	References
			Batch N	Man. date	Exp. date			
Chloroquine phosphate	Nigeria	All Tablet	NI	NI	NI	Assay (NC)	94%	(55)
Chloroquine sulfate							79%	
Quinine sulfate							24%	
Metronidazole	Nigeria	Tablet	NI	NI	NI	Assay (NC)	72%	(55)
		Suspension					100%	
Artemeter 20 mg + lumefantrine 120 mg combination	Cameroon	Tablet	NOF 2153	01.2013	11.2015	Assay (NC)	100%	(60)
			F2929	01.2012	01.2016		100%	
			F1901	01.2012	01.2014		100%	
			F2261	01.2012	01.2014		100%	
Quinine	Congo	Tablet	NI	NI	NI	Assay (NC)	100%	(54)
Albendazole 600 mg	Ethiopia	Tablet	NI	NI	NI	Assay (NC)		(58)
Chloroquine amoxycillin	Nigeria	Tab.,syrup, inj.Caps.&oral susp.	NI	NI	NI	Assay (NC)		(59)

NI, not indicated; NC, non-complaint.

### 3.5 Challenges in product regulation

#### 3.5.1 Human resource in medicines regulation

Both the shortage of well-trained health professionals and the level of motivation among available staff have been repeatedly attributed to the scarce status of the current health service to alleviate existing health problems in Africa. A severe shortage of health professionals in sub-Saharan Africa has been indicated as a major problem for scaling up the quality of service delivered to communities in these localities (71). Improving the human resource situation as a tool for health development goals is equally important to bring about change in health service quality, including service supply regulation.

The quality by design reality in the regulatory sector needs long-term vision for improvement in the government or public institutions, which will rely on the way human resources are managed in the sector. Regulatory enforcement in the health sector is inherently affected by the level of motivation among the available staff working in these public institutions. Motivations can be either financial in terms of supporting livelihoods or non-financial motivations related to the establishment of transparent institutional management that is palatable to health workers (72). Institutional transparency can be the basis for required task ownership and responsibility. In this regard sub-Saharan African countries are known for lower budgetary allocations compared to other resource rich countries (73) which can be reflected in lower financial incentives allocated to the sector.

#### 3.5.2 Law enforcement and corruption

Despite the fact that many African states have regulatory establishments and legal frameworks, they are not only less implemented but also not powerful compared to the economic benefits illegal dealers achieve from illegal trade. This has been substantiated by reports in east African economic community member states (Tanzania, Brundi, Kenya, Rwanda, and Uganda) (74).

Law enforcement in product quality regulation involves an integrated joint task including different stakeholders, such as inspectors from medicines regulation, usually of technical skills, community police units, and judicial bodies, to interpret and implement corrective actions of wrongdoers against codified legislation. However, gaps in law enforcement have been found and published in African countries, demanding attention for quality by design reality to come into effect on the ground. Among the observations (75).

Besides defective rule of law implementation, corruption is a common phenomenon. Corruption happens when an agent in service at any one of the public or private institutions can influence the expected outcome of service, enabled to give decisions on an exclusive basis, the corrupted exercise of role with an intended consequence to bring a private benefit for an agent or another affiliated person, company, organization, etc. The corrupt act usually takes place in conditions where there is a lack of transparency in the rules or concealed information. In the presence of corruption, resources are used inefficiently, with higher costs and prices resulting in distortions in output with reduced quality (76).

The impact of corruption affecting decision decisions on any one of the components in the pharmaceutical system is detrimental to health gains expected from access to quality medicines (77). In drug discovery, corruption whether with its actual or perceived impact is considered as one of the number of issues precluding pharmaceutical companies from undertaking clinical trials in Africa (78), and affecting the continent in its share of global drug development efforts. Corruption in the pharmaceutical sector (79).

In sub-Saharan Africa, studies related to corruption in the overall health sector in South Africa have indicated affecting that it negatively both patient care and healthcare worker morale (80). Regulatory costs and irregularity in budget allocation has have been indicated as an impediment to African product quality regulation (43).

#### 3.5.3 Inconsistencies in regulatory infrastructure

Africa has 54 regulatory authorities with 10 keys (81) with regulatory functions of registration licensing post-market surveillance (5). The legal framework established in product regulation differs among countries in Africa, lacking uniformity and creating problems during transboundary drug trade and import-export procedures (11). In many African countries, veterinary and medical products are regulated by sections under the health ministry, while others establish veterinary product regulation under the ministry of agriculture, as is the is the case in Ethiopia (41). Furthermore, agricultural pesticides are managed under separate sections, either from medical devices or even from veterinary pesticide regulatory units (Table 3). Living aside the pros and cons behind specialized structures to handle regulatory units under fragmented sections, harmonization of quality control schemes cannot be managed equally at the same pace. Resource redundancy in building the same facility in different sections is also not economically sound. In some countries, ownership claims have also been raised by veterinarians when veterinary pharmaceuticals have been regulated together with human medical supplies. Resource scarcity in developing countries challenges the ability to follow American and European pharmacopeias monographs (82).

**TABLE 3:** Regulatory setup in African countries as categorized by target regulated product category.

Regulated product category	Country	Regulatory body	Accountability
Veterinary medicines & pesticides	Ethiopia	EAA	MOA
Human medicinal products/cosmetics	Ethiopia	EFDA	MOH
Veterinary medicines/human medicinal products	South Africa/	SAHPRA	MOH
Agricultural pesticides	South Africa	DAFF	DAFF
Human medicinal products/veterinary products/agricultural pesticides	Nigeria	NAFDAC	MOH
Veterinary medicines	Kenya	VMD	MoA
Human medicinal products	Kenya	PPB	MOH
Agrochemicals for pest control	Kenya	Pest control products board	

Lack of integration and disorientation among hierarchical stakeholders in controlling illegal drug trade has been indicated as a problem in Ethiopian veterinary pharmaceutical quality assurance (83). African health services are known to be marred by the availability of analytic infrastructure that is needed to support the analytic quality needed in both regulatory and diagnostic procedures. New drug discovery and product development are also hampered by the level of analytic procedures and institutional credibility. The unreliability of analytic laboratory tests in Africa makes healthcare ineffective in terms of both time and expenditure (84).

#### 3.5.4 Regulatory quality reference platform

From modern global trends in product quality regulation, product quality assurance relies on officially established and agreed-upon facts and parameters from the scientific community. In this regard, pharmaceutical quality assurance relies on pharmacopeia references prepared by a group of experts, even at earlier times before the establishment of respective national regulatory bodies. For example, the initial USP compilation was prepared in 1820. Among the most common ones, USP (8) and BP in 1864 (MHRA) (85), INP (Indian Drug Control Authority), CP Chinese pharmacopeia, or pharmacopeia, in its modern sense, is a legally binding collection, prepared by a national or regional authority, of standards and quality specifications for medicines used in that country or region.

A quality specification is composed of a set of appropriate tests that will confirm the identity and purity of the product, ascertain the strength (or amount) of the active substance, and, when needed, its performance characteristics (86). Reference substances, i.e., highly-characterized physical specimens, are used in testing to help ensure the quality, such as identity, strength, and purity, of medicines. National medicines regulatory policy recommends the inclusion of Pharmacopeia used in their respective quality regulations (87). World health organizations has prepared IP and encourage member states to use it in a bid to globally harmonize regulatory schemes (WHO), the texts cover pharmaceutical starting materials, excipients, intermediates and finished pharmaceutical products (FPPs).

General requirements may also be given in the pharmacopeia on important subjects related to medicine quality, such as analytical methods, microbiological purity, dissolution testing, stability, etc. Unfortunately, no standard pharmacopeia except Egyptian pharmacopeia issued from any one of the African countries can be obtained (9). Instead, many African countries adopt any one of the popular pharmacopeias either through inclusion in their national medicines authorities quality document or informal use according to the ease of its use in a product-specific context. However, the problem arises when marginal quality findings are obtained and judgement via the use of different pharmacopeia results in different outcomes, as recommended acceptance ranges differ between pharmacopeia (88).

### 3.6 Trends and constraints for local pharmaceutical production in Africa

Low and middle-income countries in the African Region are the only group of countries in which mortality rates due to acute diseases are expected to remain in excess of those for chronic diseases, according to the World Health Organization (WHO) (89). Africa is thought to account for 73% of the AIDS-related fatalities worldwide each year. Only a lack of access to dependable medications and therapy is to blame for this intolerable human cost; thanks to advancements in modern medicine, people living with AIDS can lead happy, meaningful lives. Indeed, mortality increases when people lack access to high-quality medications (89).

Africa’s pharmaceutical business is growing because the continent’s 13 percent of people have more disposable income and are better able to make ends meet than in the past. Analysts note that between 2010 and 2020, the pharmaceutical market in Africa is expected to grow at an average annual pace of 10% (90). Together with the effects of AIDS, the main factors driving the rise of the pharmaceutical markets in Africa include the development of health insurance programs, greater investments, a better business environment, a developing regulatory framework, and growing trust in generic drugs.

The African pharmaceuticals market—excluding COVID-19 vaccines—has reached $25 billion 2022 and is expected to grow at a 6% five-year CAGR to reach $34 billion by 2027 future base-case scenario (91). This scenario is the same as estimated global pharmaceutical market growth. The implementation and/or growth of universal healthcare across the continent will lead to improved access to medicines. In the future scenario, IQVIA analysts forecast the African pharmaceutical market to reach $40 billion.

However, strong barriers to local pharmaceutical production exist across the African continent; such as, human resource constraints, inadequate infrastructure, high operating costs, weak links between local and international suppliers, and high cost of local commercial capital, poor regulation, industry fragmentation, and low production quality standards. Early experience in countries like Tanzania has shown that majority of the employees in some major drug facilities are from countries like India, due to lack of skilled local workers (92).

Insights from the Analysis of the Local Manufacturing Dynamics in Mozambique and Zimbabwe indicated that, development for local pharmaceutical manufacturing: a favorable economic outlook and support from the international community created the necessary conditions for the development of the nascent pharmaceutical industry in Mozambique, while in Zimbabwe, the presence of an established local industry was instrumental in bringing in favorable, if not always coherent, government regulation (93).

### 3.7 Trends in the life science of industry

The pharmaceutical sector in Africa is significantly underdeveloped in terms of both production capabilities and innovative practices. The continent’s pharmaceutical supply chain is heavily reliant on external funding and imports, with approximately 70% of the pharmaceutical products utilized in Africa being sourced from abroad. This industry is predominantly made up of small, privately-owned enterprises that cater primarily to their local markets. In addition to prominent multinational corporations like Sanofi and GlaxoSmithKline, which have historically maintained a robust presence in the region, a variety of drug manufacturers have recently begun to establish a notable foothold in the market (94).

Africa (kpmg.com/Africa) now hosts some of the leading global innovators and generic manufacturers. Starwin in Ghana, Saidal in Algeria, Universal in Kenya, and Aspen (one of the top 10 generic manufacturers in the world) in South Africa are home grown manufacturers. In some pockets of the continent, predominantly in North Africa and in South Africa, the status of local manufacturing of pharmaceutical products has gained a sturdy foothold (95).

In 2011, South Africa, Egypt, Algeria and Morocco accounted for more than half of the continent’s pharmaceutical sales. South Africa has a relatively well-developed pharmaceutical industry, which consists of manufacturers, distributors and dispensers forming the supply-chain (94). South African research-based pharmaceutical companies that previously belonged to either Innovative Medicines SA (IMSA) or the Pharmaceutical Industry Association of South Africa (PIASA), integrated to form a new association named the Innovative Pharmaceutical Association South Africa (IPASA) in April 2013. This created a single entity representing 25 leading pharmaceutical companies operating in South Africa. IPASA currently represents approximately 43% of the pharmaceutical private sector in the country. Overall, 37 African countries have some pharmaceutical production. Significant production capacity is being developed and enriched in Tanzania, Kenya, Uganda, Ethiopia, Ghana, and Nigeria, while Mozambique has recently commissioned an antiretroviral plant with the help of Brazil (94).

### 3.8 The pharmaceutical market in Africa and the situation of falsified and substandard medicines

The African region represents one of six WHO regions and includes 14% of the world’s population spread across 47 countries. The African region is the second most populated region with 95% of the population aged < 60 year (46). The region also faces a high (and increasing) burden of communicable diseases (CDs) and non-communicable diseases (NCDs) (46). Africa’s pharmaceutical market is growing in every sector, with a net value worth of US$28.56 billion in 2017, which has increased from a value of US$5.5 billion a decade earlier (96).

In Africa, the reliance on imported pharmaceutical products from foreign countries, coupled with quality assurance management flaws, exacerbates the issue of substandard drugs in the market (97). The issue of defective products in the pharmaceutical market is indeed a critical concern, particularly in the African industry, where poor manufacturing practices contribute significantly to the prevalence of substandard drugs (98). These defective products not only fail to meet therapeutic standards but also pose severe risks to public health, including the potential to exacerbate antimicrobial resistance and cause treatment failures. Implementing a quality-by-design (QBD) approach throughout the pharmaceutical manufacturing process can effectively address these issues. QBD emphasizes the importance of quality being built into products from the very beginning, rather than relying solely on end-product testing. This approach involves a thorough understanding of the manufacturing process and the factors that affect product quality, ensuring consistent performance throughout the product’s lifecycle (99).

### 3.9 Quality by design as solution for quality assurance

The quality-by-design concept is documented to have been coined since the time of 1992 (13) and recommended in toin the area of pharmaceutical manufacturing in 2002 after the FDA realized pharmaceutical quality assurance under conventional quality assurance inefficiency (100). Quality by design refers to a systematic approach to ensuring the quality of medicinal products by utilizing analytical, statistical, and risk management techniques throughout the various stages of design, development, and production. This concept is grounded in the examination of numerous input and process variables, necessitating a comprehensive understanding of both theoretical and analytical aspects related to these parameters (101). Besides the application of QBD in pharmaceutical manufacturing, it has also been described in the improvement of service setting as a systematic approach to design and develop a service through scientific research and quality risk management (19). QBD principles have also been defined for raw material registration (102), non-health related manufacturing activities like automobile industry (103) and non-manufacturing daily life activities like election quality assurance in USA (104) indicating its role in management of diverse human daily life activities.

The quality-by design approach includes the following components: (93). The target product or service we aspire to obtain is referred to as the quality target product profile (QTPP), which is used to define the characteristics of the final intended output, and this component of QBD helps to identify critical quality attributes (CQA) of the final output. CQA is a set of measurable characteristics for QTPP (93). The product design and knowledge of critical material attributes (CMA), which are characteristics of each input for the desired final outcome; (3) process design and knowledge of critical process parameters (CPPs), relating to CMAs and CPPs to critical quality attributes; (93) a control strategy that includes specifications for the final output, input component parts, as well as controls for steps of the production processes, often referred to as process analytical technologies (PAT); and (93) capability for processes and subsequential improvement (103).

**FIGURE 2:**
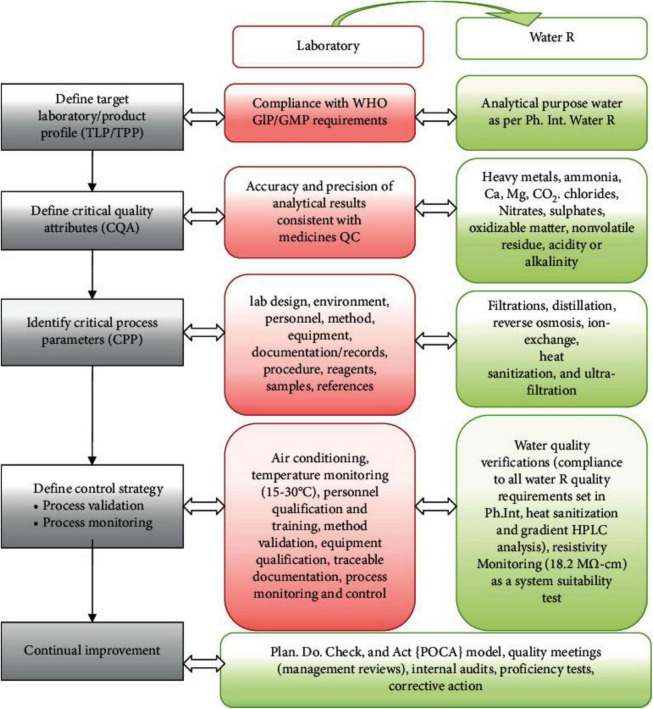
Lab QBD workflow and its application to lab water (19). GLP, good laboratory practice; GMP, good manufacturing practice.

Prior knowledge, mechanistic models, risk evaluation and analysis, quality by design experiments (DoE) and analysis of data, and process analytical technology (PAT) are all necessary QBD tools (100, 103). A study conducted by Suleman et al. (19) in Ethiopia emphasizes that the Drug Quality Control (DQC) laboratory at Jimma University was in accordance with ISO standards. This alignment was evidenced by a comprehensive assessment of quality by design (QBD) parameters, as depicted in Figure 2. A significant element of the study involved the utilization of laboratory water as a representative yet essential example of the QBD-flow, demonstrating how compliance with globally accepted laboratory water quality standards enhances the overall quality control process (19).

### 3.10 Regulatory harmonization and ICH

In the current era of pharmaceutical marketing, products in countries can be manufactured for domestic consumption and/or for export, at least to other countries. However, due to the separate regulatory authorities they owe, there are differences in regulatory procedures and customs within each country, which present difficulty and long bureaucratic procedures for manufacturers in registration and product marketing authorization (74). Besides the existing traditional way of quality assurance, no country is found to have documented the mandatory QBD procedure for marketing registration, which holds the same for all countries in the world. There are initiatives for medicine regulatory harmonization (MRH) in Africa. The SADC-MRH of the Southern African Development Community for medicines regulatory harmonization, the ECOWAS-MRH of the West African States Economic Community for medicines regulatory harmonization, and the EAC-MRH of the East African Community for medicines regulatory harmonization are underway. However, the concept of QBD, at least with theoretical concepts, is not on the agenda.

However, the regulatory harmonization committee, commonly referred to as the International Conference for Harmonization (17), was conceived in 1990 with founding members from Europe, the USA, and Japan and reformed into a non-profit legal entity in 2015, now incorporating the above 10 regulatory members. Since its establishment, it has established harmonized regulatory guidelines for quality (Q1-Q14), safety (S1-S12), efficacy (E1-E1) and multidisciplinary (M1-M15) to be used for member regulatory institutions. Among the ICH guidelines, Q8/Q9/Q10 incorporated after 2003 the concept of quality by design with better regulatory flexibility and greater room for continuous product performance improvement (105) .

Under the QBD paradigm, design space and process analytical technologies are key components. Design space is defined as the range of critical process parameters (CPP) that bring critical quality attributes (CQA) of the medicinal product within the acceptable limit, and process analytical technologies refer to the scientific tools to continuously monitor processes and output at every stage of the cycle (106). Thinking quality assurance via quality by design is therefore not easily thinkable without regulatory system harmonization. Some of the variations to be considered for harmonized quality by design perspectives therefore need to look for the following issues (Table 4).

**TABLE 4:** Comparison of pharmaceutical manufacturing by quality by design and traditional approach (103).

Component	QBD based approach	Traditional approach
Medicinal development	Empirical knowledge	Systematic multivariate experiments
Manufacturing process	Fixed	Adjustable within the design space
Control of processes	Offline analysis wide or slow response	PAT used for feedback and support for real time correction
Specification of final product	Based on batch data	Desired product performance specification
Method of control	intermediate product and finished product testing	Risk based controlled, shifted upstream, real time release
Life cycle management	Post approval changes needed	Continual improvement enabled through design space
Batch failure and recall	Reduced	To high

### 3.11 Implementing quality by design (QBD) in pharmaceutical manufacturing companies: Can the African medicine environment benefit from it?

Woodcock characterized a high-quality pharmaceutical product as one that is devoid of contamination and consistently provides the therapeutic advantages that are guaranteed on the label to the consumer (12). The US Food and Drug Administration promote risk-based methodologies and quality by design (QBD) principles in pharmaceutical development and manufacturing. This approach emphasizes embedding quality from the design phase rather than relying solely on increased testing. QBD involves understanding the manufacturing process and identifying potential risks to product quality, allowing manufacturers to implement controls that ensure consistent quality (107). This proactive strategy is considered more effective than traditional methods that focus on extensive end-product testing.

**FIGURE 3:**
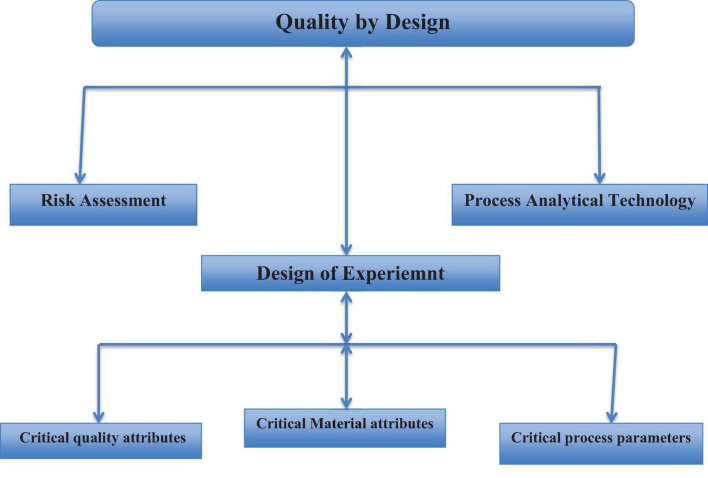
Terms and tools of quality by design in drug development.

Medicine is widely recognized as a specialized commodity, yet the advancement of the pharmaceutical industry relies heavily on innovation and production processes. Nonetheless, numerous grievances have emerged from the pharmaceutical sector regarding stringent regulations aimed at addressing defective products in the market, especially in African nations where the regulatory framework remains underdeveloped (6). The implementation of quality by design principles and methodologies in the development of pharmaceuticals in Africa is crucial for ensuring the production of defective free quality products. This is achieved through the analysis of root causes, as illustrated in Figure 3.

The pharmaceutical industry faces increased global competition and the impact of information technology, prompting a need for improved operational performance and product quality (41, 42). Key challenges include time to market, product quality, regulatory compliance, waste management, cost reduction, and cycle time. This has led to a rapid transformation in the sector, supported by regulatory authorities’ willingness to embrace innovative approaches for enhancing quality and safety. Quality by design (QBD) is now seen as essential for achieving these performance improvements (11, 72).

The advantages of implementing quality by design (QBD) in manufacturing are extensive and cover multiple dimensions (41, 42). This is especially pertinent in Africa as well as on a global scale. A comprehensive examination of the benefits associated with the incorporation of quality by design principles in the industrial sector was discussed in the current review. QBD minimizes variability and defects, leading to fewer rejected batches and less rework. This reduction in waste and inefficiency significantly cuts production costs. For instances, in several African nations, drug regulatory authorities have withdrawn a specific batch of Johnson & Johnson children’s cough syrup following reports from officials in Nigeria indicating elevated toxicity levels in that particular batch of the medication (86). This situation would not have occurred if the companies had implemented quality by design in their manufacturing practices.

By understanding the critical quality attributes (CQAs) and critical process parameters (CPPs) early in the development phase, companies can streamline their processes, reducing the time needed for product development (89). Quality by design is a systematic approach to pharmaceutical development that emphasizes understanding and controlling variability in manufacturing processes to ensure consistent product quality. However, many local manufacturers in Africa are still in the early stages of adopting these practices. As a result, the pharmaceutical markets in many African countries heavily rely on foreign imported medicines to meet their healthcare demands. This dependency on imports is often due to the higher standards of quality and reliability that these foreign products are perceived to have compared to locally produced medicines. Strengthening local manufacturing capabilities through the adoption of QBD and other advanced practices is essential for reducing this reliance and ensuring that the continent can sustainably meet its health needs with locally produced pharmaceuticals (91). This is especially pertinent in Africa as well as on a global scale. The general benefit associated with the incorporation of quality by design principles was discussed below:

#### 3.11.1 Higher operational flexibility

With improved process understanding made possible by QBD, producers may adapt their operations to changing raw material or environmental conditions without sacrificing quality (90). Companies can continuously optimize their manufacturing processes according to the methodology of quality by design (QBD) principle (93).

#### 3.11.2 Material sourcing flexibility

Diverse material sourcing: Because QBD places a strong emphasis on identifying material properties, manufacturers can get raw materials from a variety of vendors without compromising the final product’s quality (92). Because supply networks may be less dependable in African countries, this flexibility is especially important. According to a Ghanaian assessment, poorly controlled drug supply chains seriously undermine confidence, and doubt regarding the quality of medicines is not eradicated but rather handled (94). Consequently, using quality by design principles may aid in resolving these supply chain issues.

#### 3.11.3 Reduced end-process testing

Real-time quality control: By incorporating strategies for real-time monitoring and control, QBD lessens the dependence on end-process testing. This method guarantees that any problems are identified and fixed as soon as possible while speeding up the manufacturing process (95).

#### 3.11.4 Improved product consistency and robustness

Enhanced product quality: By focusing on designing quality into the product from the beginning, QBD ensures that the final product consistently meets predefined standards, resulting in safer and more effective products for patients. A report has been shown that the issue of substandard pharmaceutical products in Africa remains a significant challenge, with an estimated prevalence of 18.9% (95% CI: 14.3–23.5%) (46). Quality by design (QBD) is an essential instrument for pinpointing the root causes of quality failures in finished pharmaceutical products; however, its implementation in Africa has not yet been thoroughly explored. A study in Turkey showed that preformulating core excipients improved insights into tabletability and compatibility (97). It examined process parameters like compaction force and formulation variables such as super-disintegrant concentration within a quality by design framework. The optimized formulation was tested and validated within the established design space.

#### 3.11.5 Fewer rejected batches and rework

Increased yield: The thorough understanding and control of manufacturing processes under QBD result in fewer batch failures, leading to higher yields and reduced costs associated with rework or disposal (98). This reduced the daily quality product notifications, especially in Africa. For instance, the Ministry of Health Advisory regarding the Medical Product Alert issued by the WHO pertains to the recall of substandard pediatric medicines contain un-acceptable amount of diethylene glycol and ethylene glycol, which have been identified in the WHO Region of Africa (99). These contaminants when consumed in unacceptable amounts are detrimental to ones health.

#### 3.11.6 Faster manufacturing, testing, and approval times

Efficiency gains: Streamlined processes and reduced testing requirements lead to faster manufacturing cycles, quicker testing procedures, and more efficient batch approval processes. This speed is critical for getting products to market more rapidly (108). In Africa, the limited application of quality by design (QBD) has resulted in an extended drug registration approval process. For example, a report from South Africa revealed that the median approval time reached a lengthy 2,092 calendar days between 2011 and 2017, as determined by the Medicine Control Council’s procedures (109).

#### 3.11.7 Simplified regulatory compliance

Regulatory alignment: QBD principles align with global regulatory expectations, leading to fewer regulatory hurdles and a smoother approval process (110). The comprehensive documentation generated during QBD-based development simplifies compliance efforts.

For manufacturing companies in Africa and across the globe, the adoption of QBD can lead to more competitive operations, improve product availability, and enhance patient safety. The paradigm shift toward QBD fosters innovation, reduces dependency on reactive measures, and builds a stronger foundation for sustained quality, ultimately benefiting both manufacturers and patients worldwide (96).

## 4 Concluding remarks and future roadmaps

The review highlighted the considerable challenges faced in ensuring the availability of high-quality, safe, effective, and affordable essential medicines across African nations, aligning with the third priority objective of the global sustainable development agenda. Key obstacles include: Financial constraints limit access to quality medications, particularly in resource-poor regions. The prevalence of substandard medicines and ineffective regulatory systems was exacerbated by gaps in regulatory enforcement and poorly structured penalties for non-compliance. A regional analysis from the WHO indicated that 42% of reports concerning defective-quality medicines originate from Africa, underscoring the continent’s regulatory shortcomings. Problems like inadequate law enforcement, corruption, a shortage of human resources, and inconsistent regulatory frameworks exacerbate the complexity of product regulation in African countries. Therefore, strengthening regulatory framework, adoption of the principle of QBD, capacity building and training, financial support and investments, strengthening surveillance and reporting systems, and regional collaboration and harmonization should be taken into account to enhance the African medicine landscape.

## Authors contribution

HKH: Writing – review & editing, Visualization, Validation, Methodology, Investigation, Data curation. YTM: Writing – original draft, Writing – review & editing, Data curation, Validation, Methodology, Visualization, project administration, Investigation. AT: Writing – review & editing, Visualization, Validation, Methodology, Data curation. YO: Writing – review & editing, Visualization, Validation, Supervision, Project administration, Methodology, Data curation, Conceptualization.
