# Supplementary material for: A narrative review on problems in product quality, regulatory system constraints, and the concept of quality by design as a solution for quality assurance of African medicines
Source: Front Med (Lausanne). 2024 Oct 3;11:1472495. doi: 10.3389/fmed.2024.1472495 (PMC11484627; doi:10.3389/fmed.2024.1472495)
Supplement: Supplementary file 1 [file Table_1.DOCX]

**Supplementary file 1.** Methodological Quality assessment criteria.

| **S.N.** | **MEDQUARG** |
| --- | --- |
| 1 | The time and location of the study clearly stated |
| 2 | The definition of counterfeit or substandard medicines used mentioned |
| 3 | Type of outlets sampled |
| 4 | The sampling design and sample size calculation described |
| 5 | Type and number of dosage units purchased per outlet |
| 6 | Random sampling used |
| 7 | Information on who collected the samples |
| 8 | Packaging assessment performed |
| 9 | Statistical analysis described |
| 10 | The chemical analysis clearly described |
| 11 | Details on method validation |
| 12 | Chemical analysis performed blinded to packaging |
